# Characterization of Antioxidant and Antimicrobial Activity and Phenolic Compound Profile of Extracts from Seeds of Different *Vitis* Species

**DOI:** 10.3390/molecules28134924

**Published:** 2023-06-22

**Authors:** Luisa Pozzo, Teresa Grande, Andrea Raffaelli, Vincenzo Longo, Stanisław Weidner, Ryszard Amarowicz, Magdalena Karamać

**Affiliations:** 1 Institute of Agricultural Biology and Biotechnology—National Research Council (IBBA-CNR), Via Moruzzi 1, 56124 Pisa, Italy; luisa.pozzo@ibba.cnr.it (L.P.); teresa.grande@unifi.it (T.G.); andrea1.raffaelli@santannapisa.it (A.R.); v.longo@ibba.cnr.it (V.L.); 2 Department of Experimental and Clinical Biomedical Sciences “Mario Serio”, University of Florence, 50134 Florence, Italy; 3 Crop Science Research Center, Sant’Anna School of Advanced Studies, Piazza Martiri della Libertà 33, 56127 Pisa, Italy; 4 Department of Biochemistry, Faculty of Biology and Biotechnology, University of Warmia and Mazury, Oczapowskiego 1A, 10-719 Olsztyn, Poland; weidner@uwm.edu.pl; 5 Institute of Animal Reproduction and Food Research, Polish Academy of Sciences, Tuwima 10, 10-748 Olsztyn, Poland; r.amarowicz@pan.olsztyn.pl

**Keywords:** grape seeds, by-products, phytochemical profile, Gram-negative bacteria, Gram-positive bacteria, antiradical activity

## Abstract

Seeds of *Vitis vinifera* L. with a high content of bioactive compounds are valuable by-products from grape processing. However, little is known about the bioactivity of seeds from other *Vitis* species. The aim of this study has been to compare the phenolic composition, antimicrobial activity, and antioxidant activity of extracts from seeds of four *Vitis* species (*V. riparia* Michx., *V. californica* Benth., *V. amurensis* Rupr., and *V. vinifera* L.). Antioxidant activities were assessed as ferric-reducing antioxidant power (FRAP), 2,2-diphenyl-1-picrylhydrazyl radical (DPPH^•^) scavenging activity, and oxygen radical absorbance capacity (ORAC). The antimicrobial activity was determined using the microdilution method against some Gram-negative (*Escherichia coli*, *Salmonella enterica* ser. Typhimurium, and *Enterobacter aerogenes*) and Gram-positive (*Enterococcus faecalis* and *Staphylococcus aureus*) bacteria. Liquid chromatography-tandem mass spectrometry (LC-MS/MS) was used to evaluate the phenolic profile of extracts. Flavan-3-ols, procyanidins, phenolic acids, flavonols, anthocyanins, and stilbenoids were detected. (+)-Catechin and (−)-epicatechin turned out to be the most abundant in the phenolic profile of *V. amurensis* seed extract. Phenolic acids prevailed in the extract from *V. vinifera* seeds. The *V. riparia* and *V. californica* seed extracts had higher contents of most individual phenolics compared to the other *Vitis* species. They also showed a higher total phenolic content, DPPH^•^ scavenging activity, ORAC, and overall antibacterial activity. Total phenolic content significantly correlated with antioxidant activity and antimicrobial activity against *E. coli*. The principal component analysis (PCA) showed discrimination between *V. vinifera*, *V. amurensis*, and clustered *V. riparia* and *V. californica* with respect to variables. To recapitulate, this research demonstrates that seeds of different *Vitis* species, especially *V. riparia* and *V. californica*, are sources of molecules with antioxidant and antimicrobial activities that can be used in different sectors, such as in the food, cosmetic, and pharmaceutical industries.

## 1. Introduction

Sustainable exploitation of by-products of the food industry and underutilized food sources has become urgent due to shrinking economic margins in the food supply chain, environmental issues, and the availability of valuable yet untapped bioactive compounds [1]. Many agricultural by-products are rich in phytochemicals, like, e.g., numerous by-products of grape processing, such as pomace, stalks, sediment (residue after fermentation), and seed cake [2,3,4]. Pomace is the major by-product of wine and fruit juice production. It consists mainly of skins, seeds, and stalks, with seeds constituting as much as 38–52% of the dry weight of pomace [4]. Due to such a high share of grape seeds in the foremost waste and the widespread production of wines, the worldwide winemaking industry alone provides annually from 0.4 to 2.4 million tons of seeds for utilization [5]. Grape seeds can be managed as animal feed and as a raw material for pressing valuable dietary oil with a high content of unsaturated fatty acids, in particular, linoleic and oleic acids [6,7]. However, the seed cake left over from this process is also a by-product to be utilized. Another way is to use both grape seeds and oilcake as sources of phytochemicals, which can impart beneficial functional properties to innovative food and cosmetic products [3,4,7]. Grape seed flour and seed extracts were successfully added to confectionery and bakery products, as well as to biofilms applied as an edible coating for food storage [8,9,10,11].

The main bioactive compounds of grape seeds are phenolic compounds, which account for about 5–8% of the weight of the seeds [4]. Numerous flavan-3-ols, their oligomers and polymers, flavonols, and phenolic acids were detected in the seeds of various grapevine varieties [3,12,13,14]. Proanthocyanidins and their monomers were the most abundant [12]. Among the flavan-3-ols, both galloylated and non-galloylated compounds were identified, with (+)-catechin and (−)-epicatechin prevailing [12,13]. These monomers were most often linked by C4→C6 or C4→C8 bonds to form B-type procyanidins. In addition, anthocyanins (malvidin glucosides) and stilbenoids (*cis* and *trans* isomers of piceid, piceatannol, miyabenol C, and resveratrol) were quantified in grape seeds, but their contribution to the total phenolic content was much lower than in skins and/or stems [12,15].

Many in vitro studies have shown that phenolic compounds are responsible for the antioxidant potential of grape seeds, which is higher than that of grape skins and stems [3,16,17,18]. Positive effects of grape seed extracts on oxidative stress markers have been reported in animal models [14]. Daily doses of grape seed proanthocyanidin extracts in the range of 35–400 mg/kg of body weight of animals were sufficient to induce those protective effects. The mechanism of action of the bioactive compounds consisted of inhibiting lipid peroxidation, avoiding the production of reactive oxygen species, and thus protecting cell membranes against apoptosis. Moreover, due to their antioxidant activity, grape seed extracts may mediate the alleviation of the inflammatory process and pathologies associated with metabolic syndrome-related diseases [14,19,20]. The health-promoting properties of grape seed products have resulted in an increased interest in their use as a component of functional foods, e.g., gluten-free bread, noodles, pancakes, and cereal bars with increased antioxidant capacity [8,21]. Efforts are also being made to harness grape seed extracts as natural antioxidants in food technology. Due to their antioxidant properties, they inhibit lipid oxidation in meat products and significantly extend their shelf life [22]. Libera and co-workers reported that grape seed extract ensured similar oxidative stability of lipids in the fermentation process of pork as sodium ascorbate [23].

Globally, food spoilage caused by microorganisms still largely affects all food types and causes food waste and loss, even in developed countries. It has been estimated that the annual losses of global food reach up to 40% due to various factors, including spoilage by microorganisms [24]. Bacteria, yeasts, and molds are the common types of microorganisms responsible for the spoilage of a considerable number of food products [25,26,27]. Foodborne illnesses are another pervasive food safety problem caused by the consumption of contaminated food products, which raises a severe public health safety concern [28]. In this context, the search for strong, natural antimicrobial agents that can be used in food is a current challenge. Grape seed extracts have the potential to meet these requirements. Their antimicrobial activity against bacteria and fungi is well-established [29,30]. It is also known that the phenolic compounds present in grape seeds exhibit antimicrobial activity and are responsible for the potency of the extracts. The grape seed extracts are particularly effective against Gram-positive bacteria [12,16]. Their addition to food, e.g., meat products, has been shown to effectively reduce bacteria growth [22].

The genus *Vitis* includes about 60 species [31], but one of them—*Vitis vinifera* L.—definitely dominates grape cultivation for industrial wine production [2,4]. Other *Vitis* species, although less commonly grown, are widely distributed around the world. In China, *Vitis amurensis* Rupr. is used for winemaking [31]. American wild *Vitis* species, including *Vitis riparia* Michx. and *Vitis californica* Benth., play an important role in breeding programs aimed at obtaining hybrids with *V. vinifera* with characteristics of resistance to biotic and abiotic factors of the former [32]. Their fruits, although smaller than *V. vinifera*, have been used as food by endemic people for centuries. Currently, they are also consumed locally raw, dried, or in the form of homemade preserves. *V. californica* grows easily and is nowadays planted for riparian reclamation [33]. Despite the high prevalence and importance of wild *Vitis* species, there are few studies examining the phenolic compound profiles of their seeds [32,34,35]. Even more limited are the studies comparing seed extracts of these four *Vitis* species in terms of biological activity. A previous paper of ours reported on the reducing power and 2,2-diphenyl-1-picrylhydrazyl radical (DPPH^•^) scavenging activity of seed extracts of three wild grapevine species (*V. amurensis*, *V. riparia*, and *V. californica*) [36]; however, the extract from the cultivated species, *V. vinifera*, was not analyzed. Therefore, the aim of this research has been to find differences between the extracts from seeds of *V. vinifera* and three wild *Vitis* species (*V. amurensis*, *V. riparia*, and *V. californica*) in terms of their antioxidant activity, antimicrobial activity, and phenolic compound profile to establish whether the seeds of different *Vitis* species can be a valuable by-product after grape processing. As far as we know, the antimicrobial activity of seed extracts from different *Vitis* species has not been compared so far.

## 2. Results and Discussion

### 2.1. Total Phenolic Content

The total phenolic content (TPC) of *Vitis* species seed extracts expressed based on gallic acid equivalent (GAE) is shown in Table 1. TPC ranged from 46.6 to 121 mg GAE/g extract. *V. amurensis* seed extract had the lowest TPC, but the value determined for *V. vinifera* seed extract was not significantly different (*p* ≥ 0.05) from the former. The extracts of *V. californica* and *V. riparia* seeds had the highest (*p* < 0.05) total phenolic content.

Differences between *Vitis* species in terms of TPC of their seed extracts, i.e., *V. amurensis* < *V. riparia* = *V. californica*, were consistent with a previous study of ours [36]. However, the TPC reported for aqueous methanolic extracts of these species in the cited paper was 2.5–3.5 times higher. This could have resulted from methodological differences (e.g., results were expressed in (+)-catechin equivalents and a higher extraction temperature was applied) and from the use of a different batch of materials and reagents. A higher TPC of seeds of *V. vinifera* compared to *V. amuresnsis* was noted by Zhu and co-workers [37]. In turn, Liang and co-workers calculated the content of total phenolics as a sum of 28 individual compounds determined by HPLC-MS and found that, similar to our study, the value for *V. amurensis* seeds was lower than for *V. riparia* seeds, but *V. vinifera* seeds had a significantly higher content of phenolics among the three *Vitis* species (approximately 5 and 3 times higher, respectively) [34]. A higher TPC of seeds of *V. vinifera* (54.9 mg GAE/g) compared to wild Japanese grapevines (*V. ficifolia*, *V. coignetiae*, and *V. shiragai*) (3.6–16.5 mg GAE/g) was also reported [38]. However, it should be borne in mind that many varieties of *V. vinifera* have been cultivated, and the TPC of their seeds can vary greatly. For example, the TPC of seeds of 13 grapevine varieties grown in Serbia ranged from 38 to 103 mg GAE/g [39]; the TPC of chardonnay, concord, muscadine, and ruby red grape defatted seed flour varied between 5.93 and 89.6 mg GAE/g [6]; and the total content of extractable phenolics of seeds of grapevines cultivated in China was 5.95–13.50 mg GAE/g fresh weight (FW) [18].

### 2.2. Antioxidant Activity

The results of analyses of the antioxidant activity of grape seed extracts determined as ferric-reducing antioxidant power (FRAP), DPPH^•^ scavenging activity, and oxygen radical absorbance capacity (ORAC) are shown in Table 1. The range of FRAP values of the extracts from seeds of *Vitis* species was narrow, and a significant (*p* < 0.05) difference was found only between *V. amurensis* (with the lowest FRAP of 22.4 mg TE/g extract) and *V. riparia* (with the highest value of 35.5 mg TE/g extract). The DPPH^•^ scavenging activity of grape seed extracts ranged from 55.2 to 232 mg TE/g and varied in the following order of *Vitis* species: *V. amurensis* < *V. vinifera* < *V. californica* = *V. riparia* (*p* < 0.05). In turn, ORAC was higher (*p* < 0.05) for *V. riparia* and *V. californica* seed extracts than for *V. vinifera* and *V. amurensis* seed extracts.

The results reported for antioxidant activity are in line with those demonstrated in a previous study of ours [36], where aqueous acetonic and aqueous methanolic extracts of *V. californica* and *V. riparia* seeds showed higher DPPH^•^ scavenging activity and reduction power assayed with ferricyanide/Prussian blue than the extracts of *V. amurensis* seeds. Zhu and co-workers, in turn, showed about three times higher the average antioxidant activity of *V. vinifera* seeds compared to *V. amurensis* seeds [37]. The authors determined FRAP and antiradical activity against DPPH^•^ and ABTS^•+^ for twenty *V. amurensis* and three *V. vinifera* varieties/accessions. In another study, the antiradical activity against DPPH^•^ of *V. vinifera* seeds was within the range of the scavenging activity found for several wild *Vitis* species native to Japan (51.0 and 20.4–92.2 mmol TE/g, respectively) [38]. This was consistent with the results of the current research, although for a different set of wild *Vitis* species.

In the literature, *V. vinifera* seed extracts have been referred to as preparations with strong antioxidant properties [22,40]. Our results indicate that the extracts from other *Vitis* species, such as *V. californica* and *V. riparia,* elicited as good or even better antioxidant effects. Therefore, it seems justified to state that the seeds of these species have the potential to be a valuable material for obtaining extracts with antioxidant activity. 

The antioxidant activity of grape seed extracts significantly (*p* < 0.05) correlated with TPC (Table 2), which confirms the well-known fact that phenolic compounds are responsible for the antiradical activity and reducing power of grape seeds and their extracts [18,37,40]. Pearson’s correlation with a high correlation coefficient (0.853) was also found between DPPH^•^ scavenging activity and ORAC (Table 2). In turn, FRAP values correlated insignificantly (*p* ≥ 0.05) with ORAC values and the results of the DPPH assay. The lack of significant correlations between some assays could be due to their different underlying mechanisms [41]. In ORAC, the mode of action of antioxidants is the hydrogen atom transfer reaction; in FRAP, single electron transfer occurs; and DPPH is a mixed-mode assay. It seems that in each of these assays, other phenolic compounds may be relevant.

### 2.3. Antimicrobial Activity

**Figure 1:**
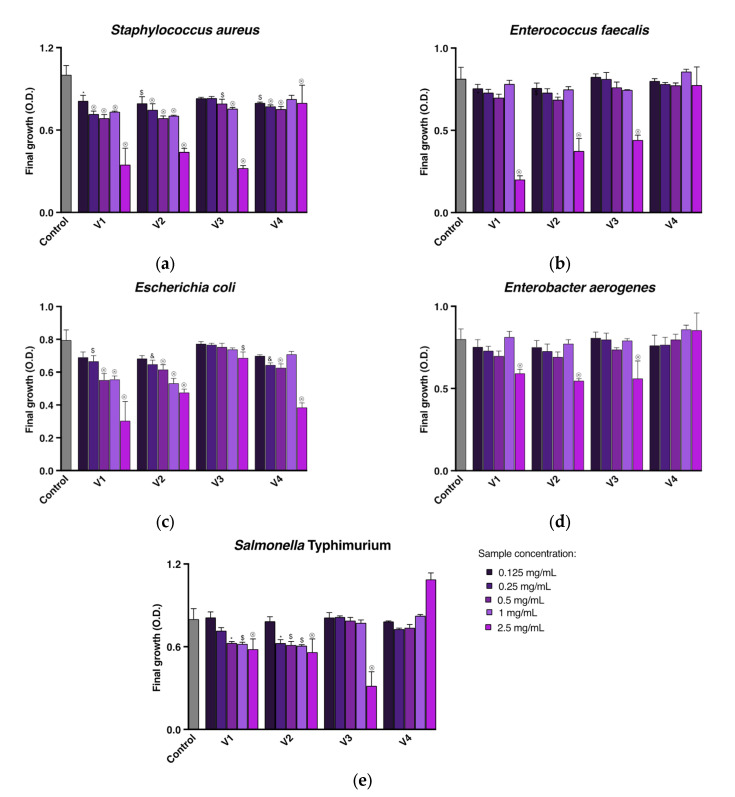
Final growth expressed as optical density (O.D.) of *Staphylococcus aureus* (**a**), *Enterococcus faecalis* (**b**), *Escherichia coli* (**c**), *Enterobacter aerogenes* (**d**) and *Salmonella enterica* ser. Typhimurium (**e**) in the presence of extracts from seeds of *Vitis riparia* (V1), *Vitis californica* (V2), *Vitis amurensis* (V3) and *Vitis vinifera* (V4) at different concentrations. Results are reported as the mean and standard deviation (*n* = 3). Sings above bars indicate a significant difference compared to control: ^⊗^ *p* < 0.0001, ^&^ *p* < 0.001, ^$^ *p* < 0.01 and * *p* < 0.05.

The antimicrobial activity of extracts from the seeds of four *Vitis* species against selected Gram-positive bacteria (*Enterococcus faecalis* and *Staphylococcus aureus*) and Gram-negative bacteria (*Escherichia coli*, *Salmonella enterica* ser. Typhimurium, and *Enterobacter aerogenes*) was determined by assessing the inhibition of bacteria growth. The results for increasing grape seed extract concentrations are shown in Figure 1. Among the Gram-negative bacteria, the final growth of *E. coli* was significantly reduced by *V. riparia* (V1), *V. californica* (V2), and *V. vinifera* (V4) seed extracts at a concentration of 0.25 mg/mL (*p* < 0.01 for V1 and *p* < 0.001 for V2 and V4) and by *V. amurensis* (V3) seed extract at a higher concentration of 2.5 mg/mL (*p* < 0.01). The growth of *S*. Typhimurium was significantly inhibited by seed extracts of *V. californica*, *V. riparia* (*p* < 0.05), and *V. amurensis* (*p* < 0.0001) at concentrations of 0.25, 0.5, and 2.5 mg/mL, respectively, while *V. vinifera* seed extract at a concentration up to 2.5 mg/mL did not show (*p* ≥ 0.05) antimicrobial activity against this bacterium. Moreover, *V. riparia*, *V. californica*, and *V. amurensis* seed extracts at the higher concentration (2.5 mg/mL) significantly (*p* < 0.0001) decreased the final growth of *E. aerogenes*. The *V. vinifera* seed extract had no significant (*p* ≥ 0.05) effect on the growth of *E. aerogenes*. The treatment of Gram-positive bacteria showed that *V. riparia*, *V. californica*, and *V. vinifera* seed extracts at low concentrations (0.125 mg/mL) significantly (*p* < 0.05 for V1 and *p* < 0.01 for V2 and V4) reduced the final growth of *S. aureus*, while the antimicrobial activity of *V. amurensis* extracts against this bacterium was significant (*p* < 0.01) at a concentration of 0.5 mg/mL. Regarding the effect of grape seed extracts on the growth of *E. faecalis*, *V. californica* seed extract caused a significant (*p* < 0.05) growth inhibition at a concentration of 0.5 mg/mL. *V. riparia* and *V. amurensis* seed extracts required a higher concentration (2.5 mg/mL) to significantly (*p* < 0.0001) reduce the final bacteria growth, while *V. vinifera* did not affect *E. faecalis* growth at any concentration tested.

Significant (*p* < 0.05) correlations were found between the antimicrobial activity of grape seed extracts against *S. aureus* and their antimicrobial activity against *S.* Typhimurium, *E. aerogenes*, and *E. faecalis* (Table 2). In addition, the inhibition rate for *E. aerogenes* correlated significantly (*p* < 0.05) with that of *S.* Typhimurium and *E. faecalis*. In turn, significant but negative correlations were noted between antimicrobial activity against *E. coli* and other bacteria (*S. aureus*, *S.* Typhimurium, and *E. aerogenes*). The TPC correlated significantly (*p* < 0.05) only with results for *E. coli*. For the other bacteria, surprisingly, the correlations were insignificant (*p* ≥ 0.05). This requires future clarification and analysis of more samples of different *Vitis* species.

A broad spectrum of antibacterial activities of *V. vinifera* seed extracts have been reported in the literature [29,30]. Several studies have shown a higher antibacterial activity of extracts from *V. vinifera* seeds against *S. aureus* than *E. coli* [12,16,42]. Our study results (Figure 1a,c) are in line with these findings. Peixoto and co-workers further found the inhibitory effect of the aqueous methanolic extract of *V. vinifera* seeds against the growth of other Gram-positive bacteria (*E. faecalis* and *Listeria monocytogenes*) and Gram-negative bacteria (*Klebsiella pneumoniae*, *Morganella morganii*, and *Pseudomonas aeruginosa*) with the minimum inhibitory concentration (MIC) in ranges of 2.5–10 mg/mL and 10–20 mg/mL, respectively [12]. Greater effectiveness against the Gram-positive bacteria compared to the Gram-negative bacteria was also noted for seed extracts of Portuguese red grapevine varieties and Bangalore blue grapes extracted by different solvents [16,40]. In the second case, grape seed extracts used at 850–1000 ppm and 1250–1500 ppm completely inhibited Gram-positive and Gram-negative bacteria, respectively [40]. Our study showed that the final growth of Gram-positive bacteria was reduced at lower concentrations than the growth of Gram-positive bacteria not only by *V. vinifera* seed extract but also by extracts from seeds of other *Vitis* species (Figure 1). However, this rule was in effect after the exclusion of *E. faecalis*. The cell wall membrane of Gram-negative bacteria is bilayered, and the outer membrane may impede the uptake of phenolics from extracts [29,30]. Therefore, Gram-negative bacteria may be more resistant than Gram-positive bacteria to the phenolic compounds of grape seed extracts, regardless of the *Vitis* species from which the seeds were obtained.

Literature data report that the factors determining the antimicrobial effectiveness of *V. vinifera* seed extracts included, on the one hand, the species of bacteria to be treated and, on the other hand, extract composition (the content of bioactive compounds), which in turn was determined by the grapevine variety, plant growth conditions, and seed extraction conditions [40,42,43]. The current study, to the best of our knowledge, has for the first time shown that *Vitis* species is also such a factor. Despite some variations described above, in general, *V. californica* and *V. riparia* seed extracts proved to be more effective antimicrobial agents than *V. amurensis* and *V. vinifera* seed extracts in inhibiting the growth of different bacteria species.

### 2.4. Phenolic Compound Profile of Seed Extracts

Liquid chromatography-tandem mass spectrometry (LC-MS/MS) was used to determine the phenolic composition of extracts from seeds of *Vitis* species. In total, 39 compounds have been detected in each sample. Their names and contents in extracts are shown in Table 3. (+)-Catechin, (−)-epicatechin, procyanidin B2, and gallic acid were the major phenolics of *V. riparia*, *V. californica*, and *V. amurensis* seed extracts, with contents in the range of 168–246 mg/100 g, 90.7–117 mg/100 g, 35.8–124 mg/100 g, and 44.6–166 mg/100 g, respectively. *V. vinifera* seed extract had a slightly lower content of (−)-epicatechin (29.4 mg/100 g) but was rich in protocatechuic acid and quercetin (39.93 mg/100 g and 23.8 mg/100 g, respectively). Noteworthy is also the high content of procyanidins B1 and B3 in the extracts. All of the compounds mentioned above are commonly determined as the major phenolic compounds of *V. vinifera* seeds, although their proportions vary greatly depending on the plant variety, seed origin, and method of analysis. [3,12,13,39,42]. The content of other phenolic acids and flavonols did not exceed 1 mg/100 g (Table 3). The exception was vanillic acid, whose content was 1.42–4.25 mg/100 g. The presence of caffeic acid and its derivatives, vanillic acid, *p*-coumaric acid, and ferulic acid, as well as quercetin and kaempferol glycosides, in grape seeds is consistent with literature data [3,16,39]. Gallic acid glucoside was not detected in our study, although it was determined to be the main phenolic acid in the seeds of *V. vinifera* as well as other *Vitis* species [12,34]. Among the anthocyanins, only the content of malvidin 3,5-*O*-diglucoside in *V. riparia* and *V. californica* seed extracts exceeded 1 mg/100 g (Table 3). This is not surprising because anthocyanins are found mainly in grape skins [16,37]. They were identified in the seeds of selected cultivars only [12,18]. Since the content of the seeds is close to a trace, the method of their detection also seems to be of importance. Nevertheless, malvidin hexoside, malvidin rutinoside, and cyanidin 3-glucoside have previously been identified in *V. vinifera* seeds [12,18]. Resveratrol has been found as the major stilbenoid of *V. vinifera* and wild grape seeds [16,34], but its glucoside has also been identified [15]. Their presence in the seeds of different *Vitis* species was confirmed by our research (Table 3).

**Figure 2:**
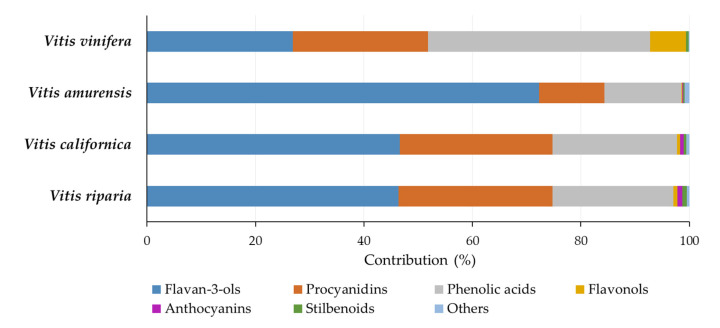
Percentage contribution of different classes of phenolics to the total phenolics of seed extracts from different *Vitis* species.

The contribution of various classes of phenolics in grape seed extracts to the total phenolics is shown in Figure 2. The proportions of phenolics in *V. riparia* and *V. californica* seed extracts were similar. For both species, flavan-3-ols, procyanidins, and phenolic acids accounted for approximately 46%, 28%, and 22%, respectively. The contributions of flavonols, anthocyanins, and stilbenoids were 0.59–0.71%, 0.65–0.94%, and 0.54–0.76%, respectively. The *V. amurensis* seed extract showed a higher prevalence of flavan-3-ols (72.32%) and lower contributions from other classes of phenolics, including procyanidins (12.06%) and phenolic acids (14.17%). Flavonols, anthocyanins, and stilbenoids occurred in a low percentage (below 0.3%). The extracts of the seeds of *V. vinifera* had a slightly different composition, with a relatively high proportion of phenolic acids (41.02%) and flavonols (6.55%) and a low contribution of flavan-3-ols (26.86%) compared to the other extracts.

The high contribution of flavan-3-ols and their dimers to the profile of phenolic compounds, as well as the dominant proportion of gallic acid among phenolic acids, was consistent with the literature data reported for seeds of *V. vinifera* varieties and their extracts. Peixoto and co-workers found that 8.3 mg/g of flavan-3-ols and procyanidins (81.4%), 1.3 mg/g of flavonols (12.7%), and 0.6 mg/g of gallic acid and gallic acid glucoside (5.9%) contributed to the total content of phenolic compounds at 10.2 mg/g in the seed extract [12]. An even lower share of gallic acid and its glucoside (1.8–3.9%) was determined by Krasteva and co-workers in the seeds of several *V. vinifera* cultivars [42]. Pantelić reported large disproportions between phenolic acids and flavan-3-ols in the seeds of Serbian *V. vinifera* cultivars; their contribution accounted for 6.02–59.9% and 36.6–92.3% of total phenolics, respectively, whereas 0.5–3.5% was noted for the share of flavonols [39]. The ratio of flavan-3-ols to procyanidins has usually been reported as approximately 1:1 [34,40,42], but a greater proportion of the former [3] as well as the latter [12] has also been shown. Liang and co-workers compared the phenolic compound profiles of seeds of 17 *Vitis* species and several accessions for each species and found that, on average, 46.9% of the phenolic compounds were flavan-3-ols, the contribution of procyanidin dimers and trimers was 35.1% and 14.4%, respectively, and the remaining compounds (gallic acid and its derivatives and flavonols) accounted for less than 4% [34]. The authors also showed that the total phenolic content was the main driver of variation among species (84.5% of the total variation); however, the variation among accessions within species was also significant [34]. Flavonols and stilbenoids were the classes of phenolics that significantly differentiated grapevines within species and accessions. In turn, while analyzing the metabolomic profiles of grapes of American *Vitis* species and *V. vinifera*, Narduzzi and co-workers concluded that flavonols differentiated the phenolics of grape skins [32]. In the seeds, *V. vinifera* varieties had a higher content of flavan-3-ols and oligomeric procyanidins than wild American grapes. The differentiating factor was also the content of gallic acid and its derivatives, the accumulation of which was higher in the seeds of American *Vitis* species, although some varieties of *V. vinifera* with a high content of compounds of this class were also found. Our study demonstrated a high contribution of flavan-3-ols to the phenolic profile of *V. amurensis* seed extracts, a high contribution of phenolic acids and flavonols to the profile of *V. vinifera* seed extract, as well as a high proportion of anthocyanins and stilbenoids in the profiles of extracts of *V. riparia* and *V. californica* seeds (Figure 2). These seem to be the main factors differentiating the *Vitis* species.

The antioxidant activity of the extracts from the seeds of the considered *Vitis* species can be attributed to the high contents of flavan-3-ols, procyanidins, and gallic acid, the more so that these compounds, especially procyanidins and proanthocyanidins, are known for their high antioxidant activity [44,45]. They are excellent free radical scavengers, and their antioxidant potential is higher than that of tocopherols and ascorbic acid. A previous study has shown that flavan-3-ols and procyanidins are responsible for the antioxidant activity of the seeds of *V. vinifera* and other *Vitis* species [35,37,38]. Flavan-3-ols and procyanidins also have high antimicrobial activity and, in addition, can act synergistically with other antibacterial agents [29,46]. Due to their ability to bind to proteins, they can inactivate bacterial enzymes, interact with cell wall proteins, transporter proteins, penicillin-binding proteins, and other surface-adhesion proteins, and consequently promote cell death [29,30]. Gallic acid may also be involved in the antimicrobial effects of grape seed extracts [30].

### 2.5. Overall Rate of Results with Principal Component Analysis

**Figure 3:**
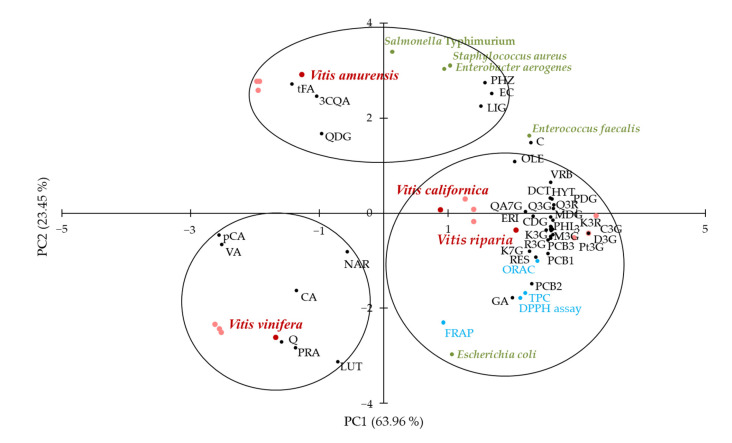
Principal component analysis (PCA) plot with distribution of the variables including total phenolic content (TPC), antioxidant activity (DPPH assay, FRAP, and ORAC), antimicrobial activity against *Escherichia coli*, *Salmonella enterica* ser. Typhimurium, *Enterobacter aerogenes*, *Staphylococcus aureus*, and *Enterococcus faecalis,* and contents of individual phenolic compounds of the *Vitis* species seed extracts. The acronyms at the black dots correspond to the names of the compounds given in Table 3. FRAP, ferric-reducing antioxidant power; ORAC, oxygen radical absorbance capacity; DPPH assay, assay with 2,2-diphenyl-1-picrylhydrazyl radical; PC1, first principal component; PC2, second principal component.

Principal component analysis (PCA) was performed to identify possible relationships between *Vitis* species and variables, i.e., TPC, antioxidant activity, antimicrobial activity, and contents of individual phenolics. The distribution of samples and variables in the PCA plot is shown in Figure 3.

The two first principal components (PC1 and PC2) explained 87.41% of the total variance. A coherent segregation between grape seed extracts was observed. There was discrimination along PC1 between clustered *V. riparia*, *V. californica*, and other *Vitis* species and along PC2 between *V. vinifera* and *V. amurensis*. The clustering of *V. riparia* and *V. californica* with TPC, antioxidant activity determined in all assays (FRAP, ORAC, DPPH), antimicrobial activity against *E. coli*, and most of the individual phenolic contents were evident. It was noteworthy that this group of variables included all detected procyanidins (B1–B3), anthocyanins (cyanidin 3-*O*-glucoside, cyanidin 3,5-*O*-diglucoside, delphinidin 3-*O*-glucoside, peonidin 3,5-*O*-diglucoside, malvidin 3-*O*-glucoside, malvidin 3,5-*O*-diglucoside, and petunidin 3-*O*-glucoside), stilbenoids (resveratrol and its glucoside), as well as most flavonols (quercetin 3-*O*-glucoside, quercetin 3-*O*-rutinoside, quercetagetin 7-*O*-glucoside, kaempferol 3-*O*-glucoside, kaempferol 7-*O*-glucoside, kaempferol 3-*O*-rutinoside). Gallic acid, the most abundant phenolic acid in *Vitis* species seed extracts, was also related to this group. However, other phenolic acids were associated with *V. vinifera* (protocatechuic, caffeic, vanillic, and *p*-coumaric acids) and *V. amurensis* (3-*O*-caffeoylquinic and *trans*-ferulic acids). The second of these was also clustered with antimicrobial activity against *S.* Typhimurium, *E. aerogenes*, and *S. aureus*. The antimicrobial activity against *E. faecalis* and content of (+)-catechin were between two clusters on the PCA plot; they were related to both groups but less so. Quercetin, whose content in the extracts was the highest among flavonols, was grouped with *V. vinifera*.

The clustering of TPC, ORAC, DPPH^•^ scavenging activity, and antimicrobial activity against *E. coli* in PCA is in line with the results of the Pearson’s correlation analysis, showing significant positive correlations among these variables (Table 2). Similarly, discrimination between antimicrobial activity against *E. coli* and against other bacteria along PC1 is consistent with significant negative Pearson correlations. In turn, the clustering of all anthocyanins and stilbenoids with *V. californica* and *V. riparia*, flavan-3-ols with *V. amurensis*, and most of the phenolic acids with *V. vinifera* confirmed our supposition mentioned above that the compounds of these classes were mainly responsible for the differentiation of the *Vitis* species phenolic profile.

## 3. Materials and Methods

### 3.1. Plant Material, Chemicals and Reagents

The experiments were conducted on *Vitis riparia* Michx., *Vitis californica* Benth., *Vitis amurensis* Rupr. and *Vitis vinifera* L. seeds supplied by Sandeman Seeds (Lalongue, France).

All standards and reagents were of analytical grade. Hexane, methanol, Folin-Ciocalteu’s reagent, gallic acid, sodium carbonate, tripyridyltriazine, Trolox, 2,2-diphenyl-1-picrylhydrazyl (DPPH) radical, 2,2′-azobis(2-amidinopropane) dihydrochloride (AAPH), phosphate buffered saline (PBS), and LC-MS/MS standards including gallic acid (GA), hydroxytyrosol (HYT), protocatechuic acid (PRA), cyanidin 3,5-*O*-diglucoside (CDG), delphinidin 3-*O*-glucoside (D3G), peonidin 3,5-*O*-diglucoside (PDG), malvidin 3,5-*O*-diglucoside (MDG), cyanidin 3-*O*-glucoside (C3G), procyanidin B1 (PCB1), petunidin 3-*O*-glucoside (Pt3G), caffeic acid (CA), procyanidin B3 (PCB3), vanillic acid (VA), malvidin 3-*O*-glucoside (M3G), procyanidin B2 (PCB2), quercetagetin 7-*O*-glucoside (QA7G), quercetin 3-*O*-glucoside (Q3G), verbascoside (VER), kaempferol 3-*O*-rutinoside (K3R), kaempferol 7-*O*-glucoside (K7G), resveratrol 3-*O*-glucoside (R3G), oleuropein (OLE), ligstroside (LIG), luteolin (LUT), eriodictyol (ERI), naringenin (NAR), and phloretin (PHL) were purchased from Sigma-Aldrich, Inc. (St. Louis, MO, USA). The other chromatographic standards, i.e., 3-*O*-caffeoylquinic acid (3CQA), (+)-catechin (C), (−)-epicatechin (EC), quercetin 3,4-*O*-diglucoside (QDG), quercetin 3-*O*-rutinoside (Q3R), *p*-coumaric acid (pCA), *trans*-ferulic acid (tFA), 2,3-dicaffeoyl-tartaric acid (DCT), kaempferol 3-*O*-glucoside (K3G), phloridzin (PHZ), resveratrol (RES), and quercetin (Q) were acquired from Extrasynthese (Genay, France). The bacterial media Mueller Hinton Broth (MHB) and Mueller Hinton Agar (MHA) were purchased from VWR (Radnor, PA, USA).

### 3.2. Extract Preparation

Seeds were ground in a coffee mill (Bosch & Siemens Hausgeräte GmbH, Munich, Germany) into powder with a particle size <0.8 mm and defatted with hexane in a Soxhlet apparatus for 6–8 h. Powdered defatted material was extracted using 80% (*v*/*v*) methanol at a solid to solvent ratio of 1:10 (*w*/*v*) at 65 °C for 15 min using an SW22 water bath (Julabo, Seelbach, Germany) [47,48]. The process was carried out three times, and the supernatants from each step were pooled. Then, the organic solvent was evaporated under vacuum at 40 °C, and the aqueous residue was freeze-dried using the Lyph Lock 6 freeze-dry system (Labconco, Kansas City, MO, USA). Three extracts were obtained for each seed type. The prepared extracts were stored at −20 °C until analysis.

### 3.3. Determination of the Total Phenolic Content

An assay with Folin-Ciocalteu’s reagent was performed to determine TPC according to the method described by Singleton and co-workers [49]. A UV/Vis Lambda 365 spectrophotometer (Perkin Elmer, Waltham, MA, USA) was used for absorbance measurement. TPC was expressed as mg of gallic acid equivalent (GAE) per g of extract.

### 3.4. In Vitro Antioxidant Activity Assays

The antioxidant activity of grape seed extracts was explored as FRAP, DPPH^•^ scavenging activity, and ORAC. The Benzie and Strain method was used for FRAP determination [50]. The FRAP reagent was prepared, and a ferric tripyridyltriazine complex was reduced to its ferrous form by aqueous solutions of grape seed extracts (1 mg/mL) exactly as in the original method. Absorbance was measured with a UV/Vis Lambda 365 spectrophotometer (Perkin Elmer) at 593 nm after 30 minutes of color development. The results were calculated based on the standard curve plotted for Trolox and expressed as mg of Trolox equivalent (TE) per g of extract.

The DPPH radical scavenging activity was determined according to the procedure reported by Boudjou and co-workers [51]. The DPPH radicals and the grape seed extracts were dissolved in methanol. After mixing both solutions at a ratio of 39:1 (*v*/*v*), the reaction mixture was left in the dark at ambient temperature for 60 min. In parallel, a mixture with methanol instead of the extract solution was prepared. Absorbance was measured at 517 nm with a UV/Vis Lambda 365 spectrophotometer (Perkin Elmer) using methanol as a blank. Trolox was used as a standard, and results were expressed as mg of TE per g of extract.

The ORAC assay was performed as previously detailed by Gabriele and co-workers [29]. Peroxyl radicals were generated by AAPH photolysis in a solution of 0.075 M phosphate buffer (pH 7.4) at 37 °C. Fluorescein sodium salt at a 0.04 mM concentration in the reaction mixture was used as a probe. Fluorescence was read using a Victor^TM^ X3 multilabel plate reader (Perkin Elmer) at 485 nm excitation and 514 nm emission wavelengths. Results were expressed as mg of TE per g of extract.

### 3.5. Determination of the Antimicrobial Activity

The bacterial strains were supplied by the American Type Culture Collection (ATCC; Manassas, VA, USA). The antimicrobial activity of *Vitis* species seed extracts was analyzed against three Gram-negative bacteria, specifically *Escherichia coli* (ATCC 25922), *Salmonella enterica* ser. Typhimurium (ATCC 14028), and *Enterobacter aerogenes* (ATCC 13048), and two Gram-positive bacteria, i.e., *Enterococcus faecalis* (ATCC 29212) and *Staphylococcus aureus* (ATCC 25923). The effect of extracts on selected bacterial growth was determined according to the procedure detailed in a previous paper of ours [52], with some modifications. The bacteria were cultured in MHB at 37 °C for 16 h and diluted to match the turbidity of the 0.5 McFarland standard. Then, bacterial suspensions contained about 1–5 × 10^5^ CFU/mL (50 µL) MHB (100 µL), and grape seed extract solutions with concentrations of 0.063, 0.125, 0.25, 0.5, 1, and 2.5 mg/mL (100 µL) were pipetted into a 96-well plate. Parallel, a control with water instead of the extract solution was added to the plate. The optical density (O.D.) was measured at 630 nm using a microplate reader (Eti-System fast reader, Sorin Biomedica, Modena, Italy) after plate incubation at 37 °C for 24 h. Results were expressed as final growth (O.D.), which referred to the final density of the bacteria incubated with or without extracts. In addition, the inhibition rate was calculated according to Equation (1). This parameter was used in the Pearson’s correlation and the principal component analyses.

Inhibition rate (%) = (100 − O.D._sample_/O.D._control_) × 100, (1)

where, O.D._sample_ is the optical density of the sample with the extract and O.D._control_ is the optical density of the control without the extract.

### 3.6. Determination of Phenolic Profile of the Extracts

Liquid chromatography (LC) separation and tandem mass spectrometry (MS/MS) detection of phenolic compounds of grape seed extracts were performed employing a 5500+ QTrap mass spectrometer with a Turbo V ion-spray source (AB Sciex, Framingham, MA, USA) connected with an Exion LC AC system consisting of two ExionLC AC pumps, an autosampler, controller, degasser, and tray (Shimadzu, Kyoto, Japan). The extract solutions were injected into a Kinetex Byphenyl column (2.1 × 100 mm, 2.6 µm particle size; Phenomenex, Torrance, CA, USA), and elution was performed in a gradient mode using (A) water containing 0.1% (*v*/*v*) formic acid and (B) acetonitrile containing 0.1% (*v*/*v*) formic acid. The gradient program was as follows: 0–10.0 min, 5–95% B; 10.0–12.0 min, 95% B; 12.0–12.1 min, 95–5% B; and 12.1–16.0 min, 5% B. The flow rate of the mobile phase was 0.4 mL/min. The MS/MS operation source parameters were as follows: nebulizer gas (GS1), 70 (arbitrary units); turbo gas (GS2), 50 (arbitrary units); curtain gas (CUR), 10 (arbitrary units); temperature, 500 °C; ion spray voltage (IS), −4500 V (for phenolics excluding anthocyanins, negative ion mode) or +5500 V (for anthocyanins, positive ion mode); entrance potential (EP), 10 V; and dwell time, 20 ms. Nitrogen was used as a collision gas. Compound-dependent parameters, including declustering potential (DP), collision energy (CE), and collision cell exit potential (CXP), were adjusted for the specific selected reaction monitoring (SRM) transition for each compound (Table S1, Supplementary Materials). Analyst 1.7.3 software and OS 1.7 software (AB Sciex) were used for data collection and processing, respectively. Qualitative confirmation was achieved using information-dependent acquisition (IDA) criteria, taking advantage of the ion trap functionalities of the 5500+ QTrap to switch from SRM to enhanced product ions (EPI), obtaining the MS/MS spectrum using a CE of 35 eV with a CE spread of 15 eV [53]. The spectra were compared with a custom-made spectral library. The data were normalized according to matrix effect and recovery percentage. The matrix effect was calculated as the peak area of the sample spiked after extraction per peak area of the standard, while recovery was calculated as the peak area of the sample spiked before extraction per peak area of the sample spiked after extraction. Calibration curves for quantitative analysis were built using a standard mix containing all the analytes at concentrations of 0.5, 1, 2, 4, 8, 16, 32, 64, 128, and 256 ng/mL.

### 3.7. Statistical Analysis

The data were reported as the mean and standard deviation of triplicates. The statistically significant (*p* < 0.05) differences between extracts were valued with the analysis of variance (ANOVA) and Tukey’s test. Pearson’s correlation analysis was conducted in order to evaluate the correlation between variables (TPC, antioxidant activity, and antimicrobial activity). In turn, the principal component analysis (PCA) was applied as the analysis of multivariate data to characterize and separate *Vitis* species in relation to the tested variables (TPC, antioxidant activity, antimicrobial activity, and contents of individual phenolics). Analyses were performed using XLSTAT software (version 2019).

## 4. Conclusions

The extracts from seeds of *V. californica* and *V. riparia* had 1.2–2.6 times higher TPC and 1.4–4.2 times higher antiradical activity than those from *V. vinifera* and *V. amurensis* seeds. All extracts showed antimicrobial activity against both Gram-negative and Gram-positive bacteria, and those from *V. californica* and *V. riparia* seeds exhibited, generally, a higher ability to inhibit bacteria growth than the extracts from the other *Vitis* species tested. It should be noted, however, that the species of bacteria significantly determined the effectiveness of the extracts as antimicrobial agents. Their particularly low concentrations were sufficient to inhibit the growth of *S. aureus*. PCA confirmed the effect of the species on TPC, antioxidant, and antibacterial activities, underscoring that *V. riparia* and *V. californica* seed extracts are especially interesting in this respect. Mainly flavan-3-ols, procyanidins, phenolic acids, flavonols, anthocyanins, and stilbenoids were detected in the phenolic profile of the extracts from seeds of *Vitis* species. Overall, the contents of (+)-catechin, (−)-epicatechin, gallic acid, and procyanidin B2 were the highest. The contribution of phenolics of various classes in the profiles of *V. californica* and *V. riparia* seed extracts was comparable to the high proportion of anthocyanins and stilbenoids compared to the other *Vitis* species. Other classes of phenolics differentiating *Vitis* species were flavan-3-ols (high contribution in the phenolic profile of *V. amurensis* seeds) and phenolic acids (dominating in *V. vinifera* seed extracts).

The study has shown the viability of not only *V. vinifera* seed extract but also extracts from other *Vitis* species, especially *V. riparia* and *V. californica*, in the food industry as functional food additives, food preservatives, and ingredients of innovative food packaging materials, as well as in the pharmaceutical and cosmetic industries due to their antioxidant and antimicrobial activity. The results of this study highlight the importance of conducting further research to screen the bioactive properties of different wild *Vitis* species and select the most suitable species for developing new products with antioxidant and antibacterial potential.

## Figures and Tables

**Table 1:** Total phenolic content (TPC), ferric-reducing antioxidant power (FRAP), DPPH**^•^** scavenging activity, and oxygen radical absorbance capacity (ORAC) of extracts from seeds of *Vitis* species.

Grapevine Species	TPC (mg GAE/g)	FRAP (mg TE/g)	DPPH Assay (mg TE/g)	ORAC (mg TE/g)
*Vitis riparia*	121 ± 12 ^a^	40.5 ± 2.7 ^a^	232 ± 21 ^a^	262 ± 20 ^a^
*Vitis californica*	97.2 ± 7.3 ^a,b^	35.5 ± 1.4 ^a,b^	221 ± 17 ^a^	252 ± 12 ^a^
*Vitis amurensis*	46.6 ± 3.0 ^c^	22.4 ± 2.0 ^b^	55.2 ± 5.6 ^c^	141.1 ± 6.2 ^b^
*Vitis vinifera*	78.7 ± 4.4 ^b,c^	35.4 ± 1.4 ^a,b^	154 ± 14 ^b^	174 ± 13 ^b^

Results were expressed as mean ± standard deviation of three replicates. Values with different superscripts (^a–c^) by column differ significantly (*p* < 0.05). GAE, gallic acid equivalent; TE, Trolox equivalent; DPPH**^•^**, 2,2-diphenyl-1-picrylhydrazyl radical.

**Table 2:** Coefficients of Pearson’s correlations between antimicrobial activity against selected bacteria, antioxidant activity and total phenolic content of extracts from seeds of different *Vitis* species.

	*E. coli*	*S. aureus*	*S*. Typhimurium	*E. aerogenes*	*E. faecalis*	TPC	FRAP	DPPH Assay
*S. aureus*	**−0.617**							
*S*. Typhimurium	**−0.838**	**0.926**						
*E. aerogenes*	**−0.643**	**0.963**	**0.895**					
*E. faecalis*	−0.021	**0.772**	0.515	**0.711**				
TPC	**0.812**	−0.103	−0.447	−0.135	0.518			
FRAP	**0.641**	−0.492	**−0.652**	−0.460	−0.028	**0.624**		
DPPH assay	**0.730**	−0.151	−0.476	−0.147	0.381	**0.867**	0.568	
ORAC	**0.601**	0.093	−0.261	0.082	**0.637**	**0.926**	0.554	**0.853**

Values in bold indicate significant correlations between variables (*p* < 0.05). Results of antimicrobial activity analyses subjected to correlation analysis were expressed as inhibition rate. TPC, total phenolic content; FRAP, ferric-reducing antioxidant power; ORAC, oxygen radical absorbance capacity; DPPH assay, assay with 2,2-diphenyl-1-picrylhydrazyl radical.

**Table 3:** Content of individual phenolic compounds in extracts from seeds of *Vitis* species (mg/100 g).

Compound Name	Acronym	*Vitis riparia*	*Vitis californica*	*Vitis amurensis*	*Vitis vinifera*
Gallic acid	GA	166 ± 32 ^a^	137 ± 18 ^a^	44.6 ± 3.3 ^b^	105.8 ± 5.1 ^a,b^
Protocatechuic acid	PRA	5.75 ± 0.38 ^b^	6.13 ± 0.13 ^b^	4.22 ± 0.18 ^b^	39.93 ± 0.83 ^a^
3-*O*-Caffeoylquinic acid	3CQA	0.102 ± 0.034 ^b^	0.0574 ± 0.0071 ^b^	0.207 ± 0.024 ^a^	0.0838 ± 0.0079 ^b^
Caffeic acid	CA	0.0864 ± 0.0063 ^a^	0.137 ± 0.026 ^a^	0.115 ± 0.023 ^a^	0.220 ± 0.090 ^a^
Vanillic acid	VA	1.418 ± 0.074 ^c^	1.56 ± 0.16 ^c^	3.33 ± 0.27 ^b^	4.25 ± 0.21 ^a^
*p*-Coumaric acid	pCA	0.495 ± 0.039 ^c^	0.537 ± 0.016 ^c^	0.835 ± 0.019 ^b^	0.960 ± 0.023 ^a^
*trans*-Ferulic acid	tFA	0.225 ± 0.058 ^b^	0.239 ± 0.049 ^b^	0.607 ± 0.036 ^a^	0.267 ± 0.034 ^b^
2,3-Dicaffeoyl-tartaric acid	DCT	0.0423 ± 0.0029 ^a^	0.0280 ± 0.0041 ^b^	0.0111 ± 0.0033 ^c^	0.0037 ± 0.0005 ^c^
**∑ Phenolic acids**		**174.45**	**145.40**	**53.89**	**151.48**
Quercetin	Q	3.76 ± 0.36 ^b^	2.03 ± 0.17 ^b^	0.215 ± 0.026 ^b^	23.8 ± 2.9 ^a^
Quercetin 3-*O*-glucoside	Q3G	0.0752 ± 0.0023 ^a^	0.0600 ± 0.0036 ^b^	0.0383 ± 0.0037 ^c^	0.0355 ± 0.0036 ^c^
Quercetin 3-*O*-rutinoside	Q3R	0.705 ± 0.020 ^a^	0.446 ± 0.011 ^b^	0.1710 ± 0.0080 ^c^	0.0865 ± 0.0047 ^d^
Quercetin 3,4-*O*-diglucoside	QDG	0.0667 ± 0.0094 ^a^	0.0910 ± 0.0084 ^a^	0.0932 ± 0.0085 ^a^	0.0766 ± 0.0043 ^a^
Quercetagetin 7-*O*-glucoside	QA7G	0.471 ± 0.010 ^b^	0.695 ± 0.022 ^a^	0.120 ± 0.017 ^c^	0.0785 ± 0.0033 ^c^
Kaempferol 7-*O*-glucoside	K7G	0.1046 ± 0.0096 ^a^	0.1099 ± 0.0082 ^a^	0.0646 ± 0.0040 ^b^	0.0747 ± 0.0027 ^b^
Kaempferol 3-*O*-glucoside	K3G	0.312 ± 0.011 ^a^	0.217 ± 0.014 ^b^	0.0414 ± 0.0026 ^c^	0.0539 ± 0.0025 ^c^
Kaempferol 3-*O*-rutinoside	K3R	0.098 ± 0.013 ^a^	0.0679 ± 0.0044 ^a^	0.0057 ± 0.0012 ^b^	0.0054 ± 0.0011 ^b^
**∑ Flavonols**		**5.59**	**3.72**	**0.75**	**24.20**
Cyanidin 3-*O*-glucoside	C3G	0.1360 ± 0.0033 ^a^	0.0699 ± 0.0065 ^b^	0.0058 ± 0.0003 ^c^	0.0038 ± 0.0003 ^c^
Cyanidin 3,5-*O*-diglucoside	CDG	0.809 ± 0.020 ^a^	0.381 ± 0.018 ^b^	0.0166 ± 0.0029 ^c^	0.0024 ± 0.0005 ^c^
Delphinidin 3-*O*-glucoside	D3G	0.433 ± 0.027 ^a^	0.3411 ± 0.0090 ^b^	0.0163 ± 0.0025 ^c^	0.0242 ± 0.0033 ^c^
Peonidin 3,5-*O*-diglucoside	PDG	1.128 ± 0.088 ^a^	0.719 ± 0.014 ^b^	0.184 ± 0.012 ^c^	0.0041 ± 0.0004 ^c^
Malvidin 3-*O*-glucoside	M3G	0.350 ± 0.041 ^a^	0.2131 ± 0.0049 ^b^	0.0179 ± 0.0017 ^c^	0.0391 ± 0.0033 ^c^
Malvidin 3,5-*O*-diglucoside	MDG	4.41 ± 0.17 ^a^	2.384 ± 0.083 ^b^	0.290 ± 0.025 ^c^	0.0170 ± 0.0033 ^c^
Petunidin 3-*O*-glucoside	Pt3G	0.1630 ± 0.0041 ^a^	0.0832 ±0.0041 ^b^	0.0022 ± 0.0005 ^c^	0.0044 ± 0.0005 ^c^
**∑ Anthocyanins**		**7.43**	**4.19**	**0.53**	**0.10**
(+)-Catechin	C	246 ± 35 ^a^	206 ± 16 ^a^	168 ± 15 ^a^	69.8 ± 3.3 ^b^
(−)-Epicatechin	EC	117 ± 15 ^a^	90.7 ± 3.4 ^a^	107.2 ± 8.4 ^a^	29.4 ± 2.5 ^b^
**∑ Flavan-3-ols**		**363.17**	**296.24**	**275.13**	**99.18**
Procyanidin B1	PCB1	51.9 ± 2.4 ^a^	40.6 ± 2.5 ^b^	4.32 ± 0.35 ^c^	13.4 ± 2.2 ^c^
Procyanidin B2	PCB2	124 ± 11 ^a^	104 ± 13 ^a,b^	35.8 ± 3.3 ^c^	69.3 ± 4.5 ^b,c^
Procyanidin B3	PCB3	46.3 ± 3.1 ^a^	34.0 ± 3.3 ^b^	5.73 ± 0.53 ^c^	9.42 ± 0.85 ^c^
**∑ Procyanidins**		**222.25**	**178.88**	**45.85**	**92.12**
Resveratrol 3-*O*-glucoside	R3G	2.72 ± 0.39 ^a^	1.87 ± 0.23 ^a^	0.437 ± 0.025 ^b^	0.652 ± 0.030 ^b^
Resveratrol	RES	3.25 ± 0.21 ^a^	1.559 ± 0.065 ^b^	0.566 ± 0.030 ^c^	1.08 ± 0.17 ^b,c^
**∑ Stilbenoids**		**6.97**	**3.43**	**1.00**	**1.72**
Hydroxytyrosol	HYT	0.560 ± 0.033 ^a^	0.378 ± 0.022 ^b^	0.144 ± 0.011 ^c^	0.0395 ± 0.0037 ^d^
Verbascoside	VER	0.219 ± 0.017 ^a^	0.1705 ± 0.0084 ^b^	0.0741 ± 0.0040 ^c^	0.0123 ± 0.0029 ^d^
Oleuropein	OLE	0.1089 ± 0.0074 ^a^	0.0504 ± 0.0029 ^b,c^	0.0638 ± 0.0036 ^b^	0.0297 ± 0.0041 ^c^
Ligstroside	LIG	0.0078 ± 0.0014 ^a^	0.0066 ± 0.0012 ^a^	0.0074 ± 0.0009 ^a^	0.0032 ± 0.0004 ^a^
Phloridzin	PHZ	2.72 ± 0.17 ^a^	2.425 ± 0.062 ^a^	2.77 ± 0.16 ^a^	0.198 ± 0.025 ^b^
Phloretin	PHL	0.0304 ± 0.0029 ^a^	0.0169 ± 0.0049 ^b^	0.0030 ± 0.0013 ^c^	0.0027 ± 0.0006 ^c^
Luteolin	LUT	0.0351 ± 0.0048 ^b^	0.0165 ± 0.0029 ^c^	0.0051 ± 0.0010 ^c^	0.0741 ± 0.0033 ^a^
Eriodictyol	ERI	0.0895 ± 0.0090 ^a^	0.101 ± 0.015 ^a^	0.0395 ± 0.0030 ^b^	0.0343 ± 0.0047 ^b^
Naringenin	NAR	0.105 ± 0.016 ^a^	0.118 ± 0.033 ^a^	0.111 ± 0.031 ^a^	0.1274 ± 0.0090 ^a^
**∑ Others**		**3.87**	**3.28**	**3.21**	**0.52**

Results were expressed as the mean ± standard deviation of three replicates. Values with different superscripts (^a–d^) by row differ significantly (*p* < 0.05).

## Data Availability

Data is contained within the article.

## Supplementary Materials

The following supporting information can be downloaded at: https://www.mdpi.com/article/10.3390/molecules28134924/s1, Table S1: The retention times (t_R_), selected reaction monitoring (SRM) transitions, and relative MS/MS parameters of phenolic compounds identified in extracts from seeds of *Vitis* species.
