# Supplementary material for: Characterization of Antioxidant and Antimicrobial Activity and Phenolic Compound Profile of Extracts from Seeds of Different Vitis Species
Source: Molecules. 2023 Jun 22;28(13):4924. doi: 10.3390/molecules28134924 (PMC10343412; doi:10.3390/molecules28134924)
Supplement: Supplementary file 1 [file molecules-28-04924-s001.zip › molecules-2467825-supplementary.pdf]

*Supplementary Materials*

# Characterization of Antioxidant and Antimicrobial Activity and Phenolic Compound Profile of Extracts from Seeds of Different *Vitis* Species

**Luisa Pozzo [1], Teresa Grande [1,2], Andrea Raffaelli [1,3], Vincenzo Longo [1], Stanisław Weidner [4], Ryszard Amarowicz [5] and Magdalena Karamać [5,*]**

[1] Institute of Agricultural Biology and Biotechnology—National Research Council (IBBA-CNR), Via Moruzzi 1, 56124 Pisa, Italy; luisa.pozzo@ibba.cnr.it (L.P.); teresa.grande@unifi.it (T.G.); andrea1.raffaelli@santannapisa.it (A.R.); v.longo@ibba.cnr.it (V.L.)

[2] Department of Experimental and Clinical Biomedical Sciences "Mario Serio", University of Florence, 50134 Florence, Italy

[3] Crop Science Research Center, Sant'Anna School of Advanced Studies, Piazza Martiri della Libertà 33, 56127 Pisa, Italy

[4] Department of Biochemistry, Faculty of Biology and Biotechnology, University of Warmia and Mazury, Oczapowskiego 1A, 10-719 Olsztyn, Poland; weidner@uwm.edu.pl

[5] Institute of Animal Reproduction and Food Research, Polish Academy of Sciences, Tuwima 10, 10-748 Olsztyn, Poland; r.amarowicz@pan.olsztyn.pl

* Correspondence: m.karamac@pan.olsztyn.pl

**Table S1.** The retention times ($t_R$), selected reaction monitoring (SRM) transitions and relative MS/MS parameters of phenolic compounds detected in extracts from seeds of *Vitis* species.

| No. | Compound Name | Acronym | Phenolic Class | $t_R$ (min) | Q1 | Q3 | DP (V) | CE (eV) | CXP (V) |
|---|---|---|---|---|---|---|---|---|---|
| 1 | Gallic acid | GA | Phenolic acid | 1.28 | 168.9 | 125.0 | −75 | −20 | −13 |
| 2 | Hydroxytyrosol | HYT | Phenylethanoid | 1.92 | 153.0 | 123.0 | −100 | −20 | −17 |
| 3 | Protocatechuic acid | PRA | Phenolic acid | 1.92 | 153.0 | 109.0 | −86 | −21 | −7 |
| 4 | Cyanidin 3,5-*O*-diglucoside (cyanin) | CDG | Anthocyanin | 2.12 | 611.1 | 287.1 | 142 | 42 | 14 |
| 5 | Delphinidin 3-*O*-glucoside (myrillin) | D3G | Anthocyanin | 2.28 | 465.1 | 303.0 | 122 | 30 | 12 |
| 6 | Peonidin 3,5-*O*-diglucoside | PDG | Anthocyanin | 2.45 | 625.1 | 301.1 | 176 | 46 | 6 |
| 7 | Malvidin 3,5-*O*-diglucoside (malvin) | MDG | Anthocyanin | 2.47 | 655.2 | 331.1 | 131 | 45 | 14 |
| 8 | 3-*O*-Caffeoylquinic acid (chlorogenic acid) | 3CQA | Phenolic acid | 2.56 | 353.0 | 191.0 | −61 | −24 | −9 |
| 9 | Cyanidin 3-*O*-glucoside (kuromanin) | C3G | Anthocyanin | 2.58 | 449.1 | 287.1 | 121 | 29 | 12 |
| 10 | Procyanidin B1 | PCB1 | Procyanidin | 2.59 | 577.1 | 289.0 | −156 | −35 | −11 |
| 11 | Petunidin 3-*O*-glucoside | Pt3G | Anthocyanin | 2.60 | 479.2 | 317.0 | 20 | 30 | 14 |
| 12 | (+)-Catechin | CT | Flavan-3-ol | 2.69 | 289.0 | 244.9 | −108 | −22 | −11 |
| 13 | Caffeic acid | CA | Phenolic acid | 2.80 | 178.9 | 135.0 | −86 | −23 | −11 |
| 14 | Procyanidin B3 | PCB3 | Procyanidin | 2.80 | 577.1 | 289.0 | −156 | −35 | −11 |
| 15 | Vanillic acid | VA | Phenolic acid | 2.86 | 166.9 | 108.0 | −62 | −26 | −13 |
| 16 | Malvidin 3-*O*-glucoside (oenin) | M3G | Anthocyanin | 2.89 | 493.1 | 331.1 | 130 | 30 | 14 |
| 17 | Procyanidin B2 | PCB2 | Procyanidin | 2.96 | 577.1 | 289.0 | −156 | −35 | −11 |
| 18 | (−)-Epicatechin | EC | Flavan-3-ol | 2.98 | 289.0 | 244.9 | −108 | −22 | −11 |
| 19 | Quercetin 3,4-*O*-diglucoside | QDG | Flavonol | 3.00 | 625.1 | 270.9 | −178 | −85 | −12 |
| 20 | Quercetagetin 7-*O*-glucoside | QA7G | Flavonol | 3.16 | 479.1 | 316.9 | −152 | −31 | −14 |
| 21 | Quercetin 3-*O*-rutinoside (rutin) | Q3R | Flavonol | 3.29 | 609.2 | 299.9 | −154 | −48 | −11 |
| 22 | *p*-Coumaric acid | pCA | Phenolic acid | 3.35 | 163.0 | 119.0 | −65 | −18 | −11 |
| 23 | Quercetin 3-*O*-glucoside | Q3G | Flavonol | 3.46 | 463.1 | 300.0 | −154 | −37 | −5 |
| 24 | Verbascoside | VER | Phenylethanoid | 3.48 | 623.1 | 160.9 | −82 | −43 | −7 |
| 25 | Kaempferol 3-*O*-rutinoside | K3R | Flavonol | 3.52 | 593.2 | 284.9 | −138 | −40 | −5 |
| 26 | Resveratrol 3-*O*-glucoside (piceid) | R3G | Stilbenoid | 3.54 | 389.1 | 227.0 | −125 | −32 | −11 |
| 27 | *trans*-Ferulic acid | tFA | Phenolic acid | 3.65 | 193.0 | 134.0 | −62 | −20 | −8 |
| 28 | Kaempferol 7-*O*-glucoside | K7G | Flavonol | 3.69 | 447.1 | 284.9 | −158 | −38 | −5 |

| | | | | | | | | | |
|---|---|---|---|---|---|---|---|---|---|
| 29 | 2,3-Dicaffeoyl-tartaric acid (cichoric acid) | DCT | Phenolic acid | 3.75 | 473.1 | 149.0 | −76 | −30 | −8 |
| 30 | Kaempferol 3-*O*-glucoside | K3G | Flavonol | 3.85 | 447.1 | 284.1 | −202 | −39 | −11 |
| 31 | Phloridzin | PHZ | Dihydrochalcone | 4.01 | 435.1 | 272.9 | −135 | −23 | −5 |
| 32 | Oleuropein | OLE | Secoiridoid | 4.09 | 539.1 | 275.0 | −137 | −32 | −12 |
| 33 | Ligstroside | LIG | Secoiridoid | 4.42 | 523.1 | 291.0 | −117 | −32 | −11.8 |
| 34 | Resveratrol | RES | Stilbenoid | 4.46 | 227.1 | 185.0 | −179 | −25 | −9 |
| 35 | Luteolin | LUT | Flavone | 4.60 | 284.9 | 133.0 | −130 | −44.6 | −17.4 |
| 36 | Eriodictyol | ERI | Flavanone | 4.60 | 287.1 | 135.0 | −121 | −34 | −10 |
| 37 | Quercetin | Q | Flavonol | 4.64 | 301.0 | 150.9 | −113 | −38 | −8 |
| 38 | Naringenin | NAR | Flavanone | 5.02 | 270.9 | 150.9 | −120 | −25 | −10.5 |
| 39 | Phloretin | PHL | Dihydrochalcone | 5.13 | 273.0 | 167.0 | −103 | −38 | −11 |

DP, declustering potential; CE, collision energy; CXP, collision cell exit potential.
